# Remunerating private psychiatrists for participating in case conferences

**DOI:** 10.1186/1743-8462-2-33

**Published:** 2005-12-18

**Authors:** Jane E Pirkis, Alan N Headey, Philip M Burgess, Harvey A Whiteford, Josh P White, Catherine Francis

**Affiliations:** 1 School of Population Health, The University of Melbourne, Melbourne, Australia; 2 School of Population Health, The University of Queensland, Brisbane, Australia

## Abstract

**Background:**

On 1 November 2000, a series of new item numbers was added to the Medicare Benefits Schedule, which allowed for case conferences between physicians (including psychiatrists) and other multidisciplinary providers. On 1 November 2002, an additional set of numbers was added, designed especially for use by psychiatrists. This paper reports the findings of an evaluation of these item numbers.

**Results:**

The uptake of the item numbers in the three years post their introduction was low to moderate at best. Eighty nine psychiatrists rendered 479 case conferences at a cost to the Health Insurance Commission of $70,584. Psychiatrists who have used the item numbers are generally positive about them, as are consumers. Psychiatrists who have not used them have generally not done so because of a lack of knowledge, rather than direct opposition. The use of the item numbers is increasing over time, perhaps as psychiatrists become more aware of their existence and of their utility in maximising quality of care.

**Conclusion:**

The case conferencing item numbers have potential, but as yet this potential is not being realised. Some small changes to the conditions associated with the use of the item numbers could assist their uptake.

## Background

In Australia, there has been growing concern that sub-optimal collaboration between different providers may impede the quality and outcomes of care, both in the physical and mental health arenas. In order to facilitate greater collaboration between providers, a series of new item numbers was added to the Medicare Benefits Schedule (MBS) on 1 November 2000, which provided remuneration for case conferences between physicians and other multidisciplinary providers. These item numbers were introduced in recognition of the fact that improved co-ordination in community settings has the potential to lead to improvements in consumer impacts/outcomes through a more flexible, efficient and responsive match between consumers' needs and services [1]. The item numbers enabled physicians to take part in discharge or community case conferences of varying lengths with other providers. As a sub-group of physicians, psychiatrists were eligible to use these item numbers to improve their collaboration with other mental health care providers, including staff of state/territory funded inpatient and community mental health services, other private mental health care specialists like psychologists, and GPs and other primary care practitioners.

The original item numbers distinguished between organising and co-ordinating a case conference (where three other providers had to be present), and participating in one (where two other providers had to be). The nature of mental health care meant that the more stringent attendance requirements associated with organising and co-ordinating a case conference could not always be met, so, on 1 November 2002, an additional set of item numbers was added, designed especially for use by psychiatrists. These new item numbers relaxed the attendance requirement for organising and co-ordinating a case conference, reducing the mandatory number of other providers to two. Table 1 provides a breakdown of the item numbers, detailing the rebate associated with the different combinations of psychiatrists' roles, number of other attendees, consumers' settings, and case conference duration.

**Table 1:** Summary of criteria for individual case conferencing item numbers

	**Provider**		**No. of other attendees**		**Role**		**Type**		**Duration in minutes**					
				
**Item No.**	**Physician (including Psychiatrist)**	**Psychiatrist only**	**3**	**2**	**Organise and co-ordinate**	**Participate**	**Community**	**Discharge**	**15–29**	**30–44**	**45+**	Fee*	Benefit (75%)*	Benefit (85%)*
820	Y		Y		Y		Y		Y			$115.55	$86.70	$98.25
822	Y		Y		Y		Y			Y		$173.40	$130.05	$147.40
823	Y		Y		Y		Y				Y	$231.15	$173.40	$196.50
825	Y			Y		Y	Y		Y			$83.05	$62.30	$70.60
826	Y			Y		Y	Y			Y		$132.40	$99.30	$112.55
828	Y			Y		Y	Y				Y	$181.80	$136.35	$154.55
830	Y		Y		Y			Y	Y			$115.55	$86.70	$98.25
832	Y		Y		Y			Y		Y		$173.40	$130.05	$147.40
834	Y		Y		Y			Y			Y	$231.15	$173.40	$196.50
835	Y			Y		Y		Y	Y			$83.05	$62.30	$70.60
837	Y			Y		Y		Y		Y		$132.40	$99.30	$112.55
838	Y			Y		Y		Y			Y	$181.80	$136.35	$154.55
855		Y		Y	Y		Y		Y			$115.55	$86.70	$98.25
857		Y		Y	Y		Y			Y		$173.40	$130.05	$147.40
858		Y		Y	Y		Y				Y	$231.15	$173.40	$196.50
861		Y		Y	Y			Y	Y			$115.55	$86.70	$98.25
864		Y		Y	Y			Y		Y		$173.40	$130.05	$147.40
866		Y		Y	Y			Y			Y	$231.15	$173.40	$196.50

* At July 2004

Source: Department of Health and Ageing [1]

The current paper reports on an evaluation of the introduction of these item numbers which aimed to: (a) examine the processes/operation of the case conferencing item numbers, from the perspective of psychiatrists and consumers; and (b) consider the costs associated with the case conferencing item numbers, and their impacts/outcomes for consumers.

## Method

### Design

The evaluation was approved by the University of Melbourne's Human Research Ethics Committee, and comprised three stages. Stage 1 examined the uptake of the case conferencing item numbers. The then Health Insurance Commission (HIC), the Australian Government agency responsible for rebates for private medical care, provided the study team with de-identified, aggregated data on the nature and extent of use of the item numbers by private psychiatrists in the three years since their introduction. The analysis considered uptake over time, but did not separately examine the first two years (in which the 'physician' item numbers were introduced) and the third year (in which the 'psychiatrist-specific' item numbers were introduced), on the grounds that the evaluation was concerned with the overall availability of an initiative that allowed psychiatrists to be remunerated for taking part in case conferences, and that the numbers available for sub-analyses would have been too small to allow meaningful conclusions to be drawn.

Stage 2 examined the impact of the item numbers on other types of services provided by private psychiatrists. The HIC identified the consumer for whom each case conference had been arranged by his/her Medicare number and, using the Medicare number, extracted information on all services provided by private psychiatrists (both those who had participated in case conferences and those who had not) for these consumers (case conferences and direct consultations). These consumers acted as their own historical controls, and their consultations in equivalent periods pre- and post- their first case conference were considered. De-identified, aggregated data were provided to the study team.

Stage 3 examined the experiences of key informants with the item numbers. Specifically, it involved interviews with private psychiatrists who had and had not used the item numbers (Stage 3a and 3b, respectively), and consumers for whom case conferences had been arranged (Stage 3c). In Stages 3a and 3b, the HIC acted as an intermediary, approaching all private psychiatrists who had made use of the case conferencing item numbers as well as a random sample of 100 who had not. The HIC sought consent from these psychiatrists for the study team to approach them for a 15-minute phone interview. Those who had used the item numbers were asked about the processes involved, the perceived impacts for themselves and their consumers regarding improvements in co-ordination of care, and whether the opportunity for case conferencing had flow-on effects in terms of better mental health outcomes for their consumers. Those who had not used the item numbers were asked about their knowledge of and attitudes towards the item numbers, why they had chosen not to use them, and whether they thought they would be likely to use them in future. Demographic details were also collected from both groups.

In Stage 3c, the psychiatrists who participated in Stage 3a were asked to act as intermediaries. Those who agreed were each sent additional information that explained Stage 3c, and asked to approach the two most recent consumers for whom they had arranged a case conference, and invite them to participate in a 30-minute phone interview. Consenting consumers were asked whether they thought that case conferencing improved the quality of care they received, and whether case conferencing had any impact on outcomes for them. Demographic details were also collected.

### Analysis

Analysis of the quantitative data in Stages 1 and 2 involved the generation of simple descriptive statistics. Analysis of the qualitative data in Stage 3 employed template analysis, which involves identifying a key set of themes which relate to specific codes, and then producing a template to organise these codes [2].

## Results

### Stage 1: Uptake of the case conferencing item numbers

**Figure 1:**
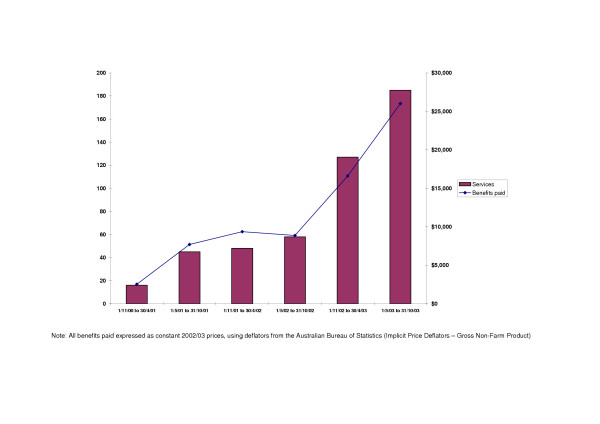
Case conferences rendered and benefits paid, by six-monthly period, 1 November 2000 to 31 October 2003 (n = 479).

The uptake of the case conferencing item numbers in the three years post- their introduction was low to moderate at best, although it did increase over time (see Figure 1). Eighty nine private psychiatrists (less than 5% of all psychiatrists) elected to use the item numbers. They rendered a total of 479 case conferences, for which the HIC paid $70,584 (around 0.01% of all services provided by and benefits paid to private psychiatrists during the period).

Table 2 shows that psychiatrists favoured the item numbers with the following characteristics: designed for use by all physicians; requiring only two other attendees to be present; requiring them to take an organisational role; facilitating community case conferences; and reimbursing for longer case conferences.

**Table 2:** Nature of case conferences rendered, 1 November 2000 to 31 October 2003 (n = 479)

**Provider**	Psychiatrist only	37%
	Physician (including psychiatrist)	63%
**Other attendees**	Two	65%
	Three	35%
**Role**	Organise and co-ordinate	72%
	Participate	28%
**Type**	Community	80%
	Discharge	20%
**Duration**	15–29 minutes	25%
	30–44 minutes	23%
	45+ minutes	51%

### Stage 2: Impact of the case conferencing item numbers on other types of services provided by private psychiatrists

Table 3 summarises the face-to-face services provided by private psychiatrists for consumers for whom case conferences had been arranged, pre- and post- their first case conference. It shows that the total number of psychiatrists involved in the consumers' care in the post- period was greater than the 89 who took part in case conferences concerning these consumers (at 163), but that this was fewer than the number involved in consumers' care in the pre-period (at 184). Despite the decrease in the total number of psychiatrists providing care from the pre- period to the post- period, the total and average number of services increased, as did the total and average benefits paid. To ensure that the latter increase was not accounted for by inflation alone, a best-case/worst-case sensitivity analysis was performed, where all of the pre- benefits paid were assumed to have been in 1997/98 dollars and converted to 2002/03 dollars, and all of the post- benefits paid were assumed to have been in 2002/03 dollars. Even under these circumstances, the post- benefits paid were higher than the pre- benefits paid.

**Table 3:** Services provided by private psychiatrists for consumers for whom case conferences had been arranged, pre- and post- their first case conference

	**Pre-**	**Post-**
**No. of providers**	184	163
**Total services**	4,861	5,499
**Average services**	26.42	33.74
**Total benefit paid – actual**	$455,743	$549,673
**Total benefit paid – adjusted**	*($511,891)*	*($549,673)*
**Average benefit paid – actual**	$2,477	$3,372
**Average benefit paid – adjusted**	*($2,782)*	*($3,372)*

### Stage 3: Key informants' experiences with the case conferencing item numbers

#### Response rates

In total, 47 key informants were interviewed. Twenty seven (30%) of the 89 psychiatrists who had used the case conferencing item numbers agreed to be interviewed, as did 16 (16%) of the 100 who had not. Three consumers and one proxy (the mother of an adolescent boy) also agreed to be interviewed. These four were drawn from a potential pool of 23 (17%), since nine psychiatrists had each agreed to recruit two consumers and a further four had agreed to recruit one.

Table 4 profiles the interview respondents, and, where possible, compares them with the sample from which they were drawn. Interviewed psychiatrists who had used the item numbers were reasonably representative of their broader group in terms of their age profile and the number of years they had been qualified as psychiatrists, but were more likely to be female and to have attained their general medical qualifications earlier. Interviewed psychiatrists who had not used the item numbers were reasonably representative of their broader group in terms of their age and sex profile, but tended to be more recently qualified (both as medical practitioners and as psychiatrists). No comparative data were available on the total sample of potential consumer interviewees.

**Table 4:** Profile of interview respondents

		**Psychiatrists who had used the case conferencing item numbers**		**Psychiatrists who had not used the case conferencing item numbers**		**Consumers for whom case conferences had been arranged**	
		**Interviewed** **(n = 27)**	**Eligible for interview** **(n = 89)**	**Interviewed** **(n = 16)**	**Eligible for interview** **(n = 100)**	**Interviewed** **(n = 4)**	**Eligible for interview** **(n = 23)**
**Age group**	<18	b	b	b	b	25%^a^	c
	18–25	b	b	b	b	0%	c
	25–34	0%	0%	0%	0%	50%	c
	35–44	19%	20%	25%	20%	25%	c
	45–54	48%	44%	31%	34%	0%	c
	55–64	30%	27%	25%	28%	0%	c
	65+	4%	6%	19%	15%	0%	c
	Missing	0%	3%	0%	2%	0%	c

**Sex**	Males	52%	67%	75%	74%	25%^a^	c
	Females	48%	29%	25%	24%	75%	c
	Missing	0%	3%	0%	2%	0%	c

**Year qualified as doctor**	pre 1960	4%	20%	13%	35%	b	b
	1960 – 1969	15%	*	25%	*	b	b
	1970 – 1979	41%	16%	19%	16%	b	b
	1980 – 1989	33%	49%	44%	33%	b	b
	1990 – 1999	7%	9%	0%	13%	b	b
	2000 – 2004	0%	*	0%	*	b	b
	Missing	0%	3%	0%	2%	b	b

**Year qualified as psychiatrist**	pre 1960	0%	4%	0%	18%	b	b
	1960 – 1969	4%	*	13%	*	b	b
	1970 – 1979	11%	*	25%	13%	b	b
	1980 – 1989	26%	27%	13%	21%	b	b
	1990 – 1999	41%	45%	50%	34%	b	b
	2000 – 2004	19%	16%	0%	12%	b	b
	Missing	0%	3%	0%	2%	b	b

a. Mother of respondent interviewed

b. Not applicable

c. Data unavailable

### Stage 3a: Experiences of private psychiatrists who had made use of the case conferencing item numbers

#### Financial incentives associated with the item numbers

Interview respondents were asked to consider their satisfaction, and often discussed this in terms of the remuneration levels offered by the case conferencing item numbers. Many commented that the item numbers provided them '*with the opportunity to be part of a decision-making forum that is not typically feasible in private practice*', because of the fee-for-service environment.

Having said this, the majority view was that the remuneration provided '*helpful compensation*', rather than covering their full costs in terms of time spent arranging or participating in case conferences. This view was exemplified in statements like: '*... I mean, you put in much more work than you are really getting paid for ... But if you are not getting anything for it, then there is a great deterrent to doing it*.'

#### Logistical issues associated with the item numbers

Respondents indicated that there were a number of logistical issues associated with the case conferencing item numbers. A number of psychiatrists noted that arranging times to meet with other care providers was '*really too much*' and should attract '*double or triple the current rate*.' This was exacerbated in circumstances where psychiatrists were using the item numbers that entailed their 'organising and co-ordinating' the case conference, rather than just 'participating' in it, because many psychiatrists assumed that the former required them to take responsibility for setting it up. In fact, 'organising and co-ordinating' refers to recording what took place during the case conference, distributing a summary of this to the treatment team, and ensuring that the consumer is informed about the outcomes of the case conference [1].

Several respondents commented on difficulties meeting the requirement that at least two other providers be present at the case conference. Sometimes, particularly in rural areas, a second party was available (e.g., a GP), but not a third. On other occasions, potential participants in the case conference did not qualify as 'formal care providers' [1] (e.g., a number of child and adolescent psychiatrists noted that teachers were integral to the team sharing responsibility for their consumer group, but did not 'count' towards the complement of other attendees).

Another observed difficulty was the fact that some providers arrived late, left early or did not attend at all. GPs were commonly cited, but there were others as well (e.g., community mental health team members). Respondents understood that these providers have hectic, unpredictable schedules, but were frustrated that their partial or non-attendance had financial implications, as indicated by the following quotation: '*There is a requirement that the specialist or GP has to be there the whole time, which is ludicrous. If someone says, "Thanks, that was good, but I've got to go now", they can't be paid for that*.' This impacted on how likely psychiatrists were to be involved in further case conferences unless the requirements changed: '*That only has to happen twice, and you begin to think it's just not worth it*.'

A number of psychiatrists cited travel time as a problem. Even those who were positive about the item numbers noted that they often felt hesitant to attend case conferences because of the time involved in getting to the meeting: '*I see a lot of people from a long way away. I could travel up to one hour one way to be involved in it. That is a disincentive for me doing this kind of thing*.'

Several psychiatrists expressed frustration at having '*claims rejected*' by the HIC. In their eyes, these rejections often occurred for minor reasons, and had a major impact on the likelihood that they and their colleagues would take part in future case conferences.

Some psychiatrists indicated that they and their colleagues had experienced confusion over some of the conditions associated with the item numbers. For example, there was a lack of clarity regarding the term 'formal care providers of a different discipline', creating confusion about whether other physicians (e.g., paediatricians) could be included in the total number.

#### Impact of case conferences on psychiatrists' roles

According to respondents, case conferencing allowed them to take on a consultant role, permitting them to offer specialist advice. They indicated that both parties benefited from this role. They enjoyed the break from direct care delivery, and appreciated meeting with colleagues working in the field, making statements like, '*Just to have more contact with GPs and other mental health professionals is really rewarding*.' They also felt that other providers (particularly GPs) were better equipped to deliver optimal care, suggesting that '*It educates the GP and helps the management of other patients ... the GP is going to use the knowledge gained in a case conference to help manage patients for the foreseeable future*.'

#### Impact of case conferences on co-ordination of care

Overwhelmingly, respondents indicated that case conferences improved co-ordination of care, particularly to consumers with complex needs and/or problems in multiple areas of life – e.g., those with a dual diagnosis of mental illness and intellectual disability, those with chronic mental health problems and/or problems that were not responsive to routine drug treatments, and children and adolescents. As one provider put it, '*... with these multiple problems, there is often a need for ... lots of other providers case conferencing is most useful in this situation*.'

A number of psychiatrists noted that case conferencing facilitated information transfer between providers, allowing for '*speedy communication and the understanding of all involved*.' They felt that being able to sit down with other providers face-to-face enabled them to clarify the problem and develop an action plan which everybody agreed upon. Psychiatrists reported that case conferencing had clear advantages over trying to communicate with other providers either by phone or through letters, and often constituted the first opportunity for all relevant providers to meet. Some went as far as to say, '*There really wouldn't be any other way of communicating with everyone if we hadn't done a case conference*.'

Respondents noted that case conferences clarified the roles and responsibilities of each provider, and helped them to perform these roles optimally. They cited examples such as, '*... in one case, the social worker had skill in the treatment of trauma and once I realised this I was able to hand over this aspect of management to the social worker*.'

Respondents observed that the case conferencing item numbers had a direct impact on the quality of assessment and treatment. With regard to assessment, respondents reported that communicating with other providers at a case conference enhanced their understanding of the consumer. Consumers with complex needs often have problems in multiple domains of life, and several psychiatrists described how case conferences aided their assessment capacity because other attending providers could give them better insight into consumers' strengths and weaknesses. With regard to treatment, many respondents commented that treatment planning was improved by including '*different views from different people*', although *s*ome added the caveat that they had to be careful to weigh up the relative merits of suggestions offered by different participants.

#### Impact of case conferences on consumer outcomes

The majority of respondents indicated that case conferences impacted positively on consumer outcomes. Some had difficulty in 'pinning down' exactly how this occurred, remarking that the case conference helped them to gain a more comprehensive understanding of different facets of the consumer's life, which translated into improved treatment and greater consumer satisfaction. One psychiatrist summarised this in the following way, '*Whatever helps me understand the person, and their treatment network, helps the patient. The patient is often not going to know how it's helped, but it gives me a broader understanding and I can use that in different ways. That is psychiatry!*'

Others were more specific, citing tangible outcomes that they believed to be a direct result of case conferencing. For example, several psychiatrists reported that a case conference had enabled the treatment team to improve management when the consumer was in crisis and/or required hospitalisation, providing examples like the following: '*We had a case conference and it worked very well ... in fact that was one and half years ago, and the patient has not had an admission since, or a crisis contact with the GP. So it has saved money*'

Respondents were also asked to consider whether case conferences had the potential to lead to negative outcomes, and the majority did not believe this to be the case. Most reported that they engaged in several practices to minimise the likelihood of this occurring, such as explaining the nature of the case conference beforehand, inviting the consumer to attend, and reviewing the process with him or her afterwards. Several noted that some consumers felt intimidated by their treatment providers meeting to discuss them, especially if they were not present at the case conference, but that this was usually resolved by offering reassurance.

### Stage 3b: Experiences of private psychiatrists who had not made use of the case conferencing item numbers

#### Reasons for non-use of the item numbers

The reasons for respondents' non-use of the item numbers were explored. As a first step, respondents were asked whether they were aware of the case conferencing item numbers before they were contacted by the study team, and, if so, whether they knew how they operated. Six respondents (37%) were unaware of the item numbers, and a further six (37%) were unsure about how they worked. In total, then, 12 respondents (75%) had insufficient knowledge of the item numbers to make use of them.

Even some of those with a basic understanding of the existence and operation of the item numbers were not fully cognizant of the conditions associated with them, and this had contributed to their non-use of the item numbers. To illustrate, one psychiatrist believed that all providers had to be physically present at the case conference. He wanted to link up with the other providers by teleconferencing, but he did not believe this was acceptable under the conditions of the item numbers and had consequently not used them. In fact, teleconferencing is permissible [1].

Those respondents who were aware of the existence and operation of the item numbers had elected not to use them for a variety of reasons. For some, the item numbers were not seen as relevant, either because their private practice was so small that involvement in case conferences was not practical, or because they saw very few consumers with complex needs for whom case conferences would have been warranted.

Some respondents indicated that they had taken part in case conferences but had not billed for them, because it had not occurred to them to do so. One psychiatrist who had attended case conferences but not charged for them was asked why and replied, '*Laziness! They are simply not in the forefront of my mind. I have done them, but I have not charged for them. It is the secretaries that have to do the billing. I simply have not mentioned it to them, and if you don't talk to them they can't charge for them*.'

Others felt that organising a case conference in a manner that satisfied the HIC's criteria was time consuming and difficult, and that the remuneration offered was incommensurate with this effort. This view is summarised by one psychiatrist who said, '*It's just too hard to arrange a time to meet with people, then plan it and run it. The payment you get for that does not compensate for the time you spend arranging it*.'

Several respondents reported that they used alternatives to case conferencing to communicate with other providers. Often this involved contacting other professionals '*bit by bit*', either by telephone or via letter, or using a single service provider as a conduit for communicating with the rest of the team.

#### Likelihood of future use of the item numbers

Respondents were asked about the likelihood that they would use the case conferencing item numbers in future. Five (31%) indicated that the item numbers were not relevant to their practice and were therefore unlikely to use them in the future.

Respondents for whom the item numbers were relevant fell into three camps concerning the issue of future use. In the first group were three psychiatrists (19%) who perceived that the remuneration provided by through the item numbers did not outweigh the logistical difficulties involved in case conferencing. They preferred to continue to use their current '*bit by bit*' model of contact with other providers.

A second group of six psychiatrists (33%) indicated that they would use the item numbers subject to checking the conditions associated with their use. For example, one psychiatrist in a rural area commented, '*Yes, I'm likely to use it provided the payment structure reflects the extra difficulties of meeting in person for rural health professionals. I'm talking about travel time, long distances etc*.'

Finally, two psychiatrists (13%) indicated that they would definitely use the item numbers in the future, having been made aware of their existence and operation. One of these psychiatrists stated, '*Since this has been brought to my attention, and more importantly to my office manager's attention, we'll bill more for this. I'll do it in the future*.'

### Stage 3c: Experiences of consumers for whom case conferences had been arranged

#### Attendance by consumers at case conferences

Three respondents had been present at the case conferences convened to co-ordinate their care. Of these, two attended face-to-face, and one attended via video link. All were positive about their involvement, indicating that it '*allowed them to be heard*.' One consumer did not attend, and was angry about this because she wanted to have input into the meeting. However, she was ultimately pleased with the outcome of the case conference.

#### Impact of case conferences on co-ordination of care

Three respondents reported that, in their experience, case conferences enhanced co-ordination of care. In particular, they noted that case conferences allowed providers to clarify their treatment roles. Respondents reported that clearing up confusion at the '*provider end*' improved clarity at '*their end*.' One respondent explained this in the following way: '*I suppose that the lines became clearer as to what everyone's job was*.'

Respondents also indicated that case conferences improved communication between all parties, helping providers to develop an appreciation of each other's points of view and work as a team. As one respondent put it, '*I found them really valuable. Having all the professionals there gave an open forum for me to present my views to them ... It meant that other professionals could also have input. Everyone was working together as a team*.'

#### Impact of case conferences on consumer outcomes

Respondents were asked whether they noted changes in their 'health and wellbeing' following the case conference, and gave mixed responses. Two expressed the view that although their coordination of care had improved, this had not impacted on their day-to-day functioning. A third, the parent of the adolescent boy, said that the case conferences arranged for her son made a small but significant improvement to his welfare, describing them as '*a contributing factor*.' The fourth respondent reported that she experienced substantial improvement in functioning as a result of the case conference. She indicated that the case conference was a powerful therapeutic event for her, although she was not specific about the mechanism by which change occurred: '*For twelve years I was in a dysfunctional relationship with physical and mental abuse. At the case conference it all just came out ... Since the case conference I'm doing things now and leading a normal life. I'm not stressed out and I'm not agoraphobic like I was*.'

## Discussion

### Study limitations

Two study limitations should be borne in mind in interpreting the above findings. The first relates to the quantitative data provided by the HIC for Stages 1 and 2. The HIC provided these data at an aggregated level, in order to protect the confidentiality of individual psychiatrists and consumers. This was entirely appropriate, but it limited the analyses that could be performed, particularly since the relatively low level of uptake of the item numbers meant that the overall numbers were small.

The second limitation relates the use of intermediaries to recruit interview participants. Again, this was considered proper practice, since it meant that the study team could not identify any psychiatrist or consumer unless they expressly consented to be interviewed. However, it placed constraints on the study team's control over the recruitment process, which undoubtedly affected the response rates. In spite of a second invitation letter being sent from the HIC to psychiatrists, and reminder calls being made to psychiatrists to encourage them to recruit consumers, the response rates for Stage 3 were relatively low.

### Interpreting the findings

#### Patterns of uptake

Despite the above caveats, the study provides useful evaluative information about the case conferencing item numbers. The key finding – that although the uptake of the item numbers has been slow, psychiatrists who have used them have been extremely positive about them, citing benefits for them and their consumers (particularly those with complex needs and/or multiple providers) – is consistent with the small amount of related work that has been done in this area in Australia. Evaluations of initiatives designed to expand the activities in which psychiatrists and GPs could be involved (the Partnership Project and the Enhanced Primary Care MBS item numbers, respectively) found that although relatively few providers took advantage of the opportunity to be involved in and remunerated for case conferences, those who did found them professionally satisfying and believed that they improved co-ordination of care for consumers with complex needs [3-11].

The fact that the uptake of the item numbers has increased over time is also consistent with the above evaluations [3-11], suggesting that any initiative of this kind may have a 'settling in' period. It would be anticipated that this increase in uptake might continue, perhaps plateauing at a certain point. It was beyond the scope of the study to monitor the uptake in a formal way for longer than three years, but informal analysis of the psychiatrist-specific item numbers (via publicly available data accessible from the HIC website) suggests that the uptake of this subset of item numbers has continued to grow. These data show that 44 case conferences were billed against the psychiatrist-specific item numbers in the first two quarters of 2003, 140 in the second two quarters of that year, 134 in the first two quarters of 2004, 256 in the second two quarters of that year, and 289 in the first two quarters of 2005. Data on psychiatrists' use of the more general physician numbers were not available from the HIC website, because the publicly accessible statistics do not allow psychiatrists to be distinguished from other physicians.

It was clear from interviews with psychiatrists who had not used the item numbers that a significant number were not aware of their existence, and even those who were expressed confusion about their operation. Once their awareness was raised, many indicated an intention to use them in the future. Having said this, there will always be some for whom the item numbers are perceived as not relevant, too stringent in their conditions, and/or not associated with sufficient levels of reimbursement.

#### Relative popularity of different item numbers

The fact that some item numbers were more popular than others is worth considering in detail. More case conferences were billed against the item numbers designed for all physicians (including psychiatrists) than against the psychiatrist-specific item numbers, a difference that is most likely to be explained by the fact that the former existed for two years before the latter were introduced. Indeed, further analysis of the data revealed that in the last year, when both types of item number were available, 57% of all claims were made against the psychiatrist-specific item numbers.

Psychiatrists more commonly elected to use the item numbers that require attendance at the case conference by two other providers, rather than those that require three others to be present. It makes intuitive sense that case conferences with two other providers might have more intrinsic appeal, given the logistical difficulties in co-ordinating meetings.

Psychiatrists were also more inclined to bill against item numbers which recognised their role in organisation and co-ordination of the case conference, as opposed to participation only. There may be several reasons for this. One might be that 'taking charge' of the case conference fits with their consultant role. Another might be that they are relatively professionally isolated, and may therefore have greater imperatives for calling together others involved in a consumer's care than, for example, members of a public sector mental health team. A third reason may relate to availability of billing options – two thirds of the item numbers allow for organisation and co-ordination, compared with only one third that allow for participation. A final reason may involve the relatively greater level of remuneration associated with organising and co-ordinating the case conference.

Community case conferences were more common than discharge case conferences. This difference may reflect the profile of the population of consumers who see private psychiatrists – most will be dwelling and functioning in the community most of the time; only some will require inpatient admissions, and even these may be infrequent. It may also reflect the fact that, when consumers are discharged from public sector inpatient units, the private psychiatrist may not always be notified, so the opportunity for setting up a case conference may be missed.

Case conferences most commonly lasted 45 minutes or longer, presumably relating to the fact that sharing information and co-ordinating care requires time, particularly if a number of providers are involved.

#### Increased, not decreased, levels and costs of overall care

The fact that the introduction of the item numbers was associated with increased, rather than decreased, levels and costs of overall care warrants further exploration. Inflation can be ruled out, since the total number of services increased and all costs were expressed in constant 2002/03 prices. However, there might be increases in the overall level of services provided by psychiatrists, at least initially, because they could continue to see consumers individually as well as being involved in case conferences. Alternatively, psychiatrists may be more likely to render case conferences for consumers whose needs are becoming increasingly complex, and who are requiring greater professional input.

It should also be noted that the study could only capture care provided by private psychiatrists and paid for (primarily at least) through the HIC, and not care delivered by other providers and paid for from other sources (e.g., care provided by staff of mental health services whose salaries are paid from state/territory health budgets). There may have been a reduction in the need for some of these services if care became more co-ordinated as a result of case conferences.

Although the introduction of the case conferencing item numbers was not associated with the hypothesised reduction in the level and cost of services by private psychiatrists, this is not necessarily a negative finding. If the increases in the quality of care outweigh the increases in costs, they may still be cost-effective. Findings from the interviews with psychiatrists who had used the item numbers and consumers for whom case conferences had been rendered suggest that this may be the case.

#### Improved quality of care

The above notion of 'improved quality of care' warrants further exploration. It is fair to say that the case conferencing item numbers improved continuity of care (' [the] ability to provide uninterrupted, coordinated care or service across programs, practitioners, organisations and levels over time' [12]). This message consistently emerged from the interviews with psychiatrists and consumers. It is less clear whether the item numbers demonstrated effectiveness (' [the] care, intervention or action achieves [the] desired outcome in an appropriate timeframe' [12]). Interview respondents expressed mixed views in this regard. Some psychiatrists indicated that case conferences had improved outcomes for consumers, although most were unable to be specific about the nature of these outcomes. Some consumers suggested that the case conferences had resulted in improvements in their day-to-day functioning, but others felt that no change had occurred.

## Conclusion

The introduction of the MBS case conferencing item numbers has not been met with overwhelming enthusiasm by psychiatrists. Psychiatrists who have used the item numbers are generally positive about them, as are consumers. Psychiatrists who have not used them have generally not done so because of a lack of knowledge, rather than direct opposition. The use of the item numbers is increasing over time, perhaps as psychiatrists become more aware of their existence and of their utility in maximising quality of care.

If the case conferencing item numbers are to achieve their potential, some consideration might need to be given to issues of process and structure. The degree of awareness of the item numbers is sub-optimal. There is confusion over some of the conditions associated with the item numbers (e.g., the definition of the term 'organise and co-ordinate', the nature of the other providers required to attend the case conference, the required duration of attendance by other providers), and there are some stipulations which made it difficult for psychiatrists to make use of the item numbers in particular circumstances (e.g. psychiatrists in rural and remote areas find it difficult to satisfy the requirement of at least two other providers being present, child and adolescent psychiatrists are concerned about the exclusion of teachers from the list of 'eligible' attendees). Finally, there are issues concerning the level of remuneration that the item numbers attract, given the amount of time required to organise and participate in them. The Australian Government may wish to take these findings 'on board' in future iterations of the MBS.

## Declaration of competing interests

The authors declare that they have no competing interests.

## Authors' contributions

JP and PB conceptualised the evaluation and took the lead on designing the study; AH assisted with refining the study design. JP liaised with the HIC in extracting the data for Stages 1 and 2. JP and AH developed the interview schedules for Stage 3, with assistance from PB, JW and CF. JP and AH undertook the data management and analysis activities associated with all three stages. JP and AH took primary responsibility for drafting the original version of the paper, and all other authors contributed substantially to revised drafts. All authors read and approved the final manuscript.
